# Dexmedetomidine: What’s New for Pediatrics? A Narrative Review

**DOI:** 10.3390/jcm9092724

**Published:** 2020-08-24

**Authors:** Mohamed Mahmoud, Egidio Barbi, Keira P. Mason

**Affiliations:** 1 Department of Anesthesia, Cincinnati Children’s Hospital Medical Center, University of Cincinnati, Cincinnati, OH 45229, USA; Mohamed.Mahmoud@cchmc.org; 2 Institute for Maternal and Child Health, IRCCS Burlo Garofolo, 34137 Trieste, Italy; egidio.barbi@burlo.trieste.it; 3 Department of Medicine, Surgery, and Health Sciences, University of Trieste, 34137 Trieste, Italy; 4 Department of Anesthesiology, Critical Care and Pain Medicine, Harvard Medical School, Boston Children’s Hospital, Boston, MA 02115, USA

**Keywords:** pediatric, anesthesia, sedation, pharmacology, neurotoxicity, neuroprotection, side effects

## Abstract

Over the past few years, despite the lack of approved pediatric labelling, dexmedetomidine’s (DEX) use has become more prevalent in pediatric clinical practice as well as in research trials. Its respiratory-sparing effects and bioavailability by various routes are only some of the valued features of DEX. In recent years the potential organ-protective effects of DEX, with the possibility for preserving neurocognitive function, has put it in the forefront of clinical and bench research. This comprehensive review focused on the pediatric literature but presents relevant, supporting adult and animal studies in order to detail the recent growing body of literature around the pharmacology, end-organ effects, organ-protective effects, alternative routes of administration, synergetic effects, and clinical applications, with considerations for the future.

## 1. Introduction

Despite the lack of pediatric labelling worldwide, the experience and enthusiasm for dexmedetomidine (DEX) for the pediatric population continues to grow as more established treatment regimens describe its use for pediatric procedural sedation, as an adjunct to attenuate emergence agitation, as a premedication for anxiolysis, as an adjunct to anesthesia (general and regional) and, most recently, for pediatric palliative care.

This narrative review was intended to provide an update of the most recent contributions to the DEX literature and will focus on the recent growing body of pediatric literature examining pharmacology, end-organ effects, organ-protective effects, alternative routes of administration, synergetic effects, and pediatric post-procedural applications [1]. Searches on PubMed, Cochrane Library, Medline, and Google Scholar were conducted for relevant literature using the terms “dexmedetomidine” and “pediatric”. Results were limited within database to published studies from 2013 to 2020 in population: 0–18 years. Searches were restricted to publications in English. Publications concerning adults and animal studies were included when judged as relevant. Randomized controlled trials, observational studies, retrospective studies, meta-analyses, abstracts, and case reports were included.

As the application of DEX for adults as well as bench and animal-related research began long before the administration in children, this paper also drew on such relevant nonpediatric literature.

## 2. Updates on Pharmacology

**Figure 1:**
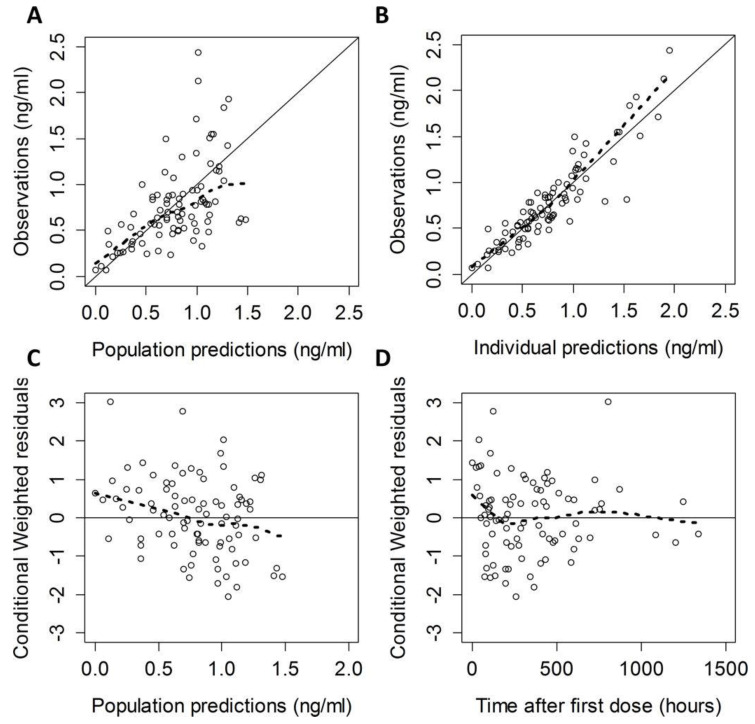
Final population pharmacokinetic (PK) model diagnostic plots: observed versus population prediction (**A**) and individual prediction (**B**), conditional weighted residuals versus population predictions (**C**), and time after last dose (**D**). The solid line in (**A**,**B**) is the line of identity. The solid line in (**C**,**D**) is a reference line at y = 0. The dashed lines in (**A**–**D**) are smooth lines.

There is an increasing body of literature examining the pharmacokinetics and pharmacodynamics of DEX for the infant and pediatric population. A dose escalation study on two groups of intubated full-term neonates (1 day–1 month and 1 month–24 months) following cardiac surgery used a nonlinear mixed effect modeling (MEM) and two-compartmental approach with intravenous DEX administered as a bolus followed by an infusion. DEX clearance was demonstrated to be significantly decreased at birth with subsequent increased clearance over the first month. Age, weight, duration of cardiac bypass, and presence of an intracardiac shunt were factors that contributed to clearance [2]. In a phase I PK study of DEX in infants, clearance and volume of distribution were 0.87–2.65 L/kg/h and 1.5 L/kg, respectively, for infants with postmenstrual age of 33–61 weeks and body weight of 2–6 kg. (Figure 1) [3].

A small pilot study in term neonates examined scaled pharmacokinetic models that were obtained by applying allometry and maturation to already published data. The clearance of intravenous DEX using this method was up to 20% higher than in previously published reports, with a clearance of 2.29 L/h in a full-term infant and higher in those born earlier [4].

The underestimation of DEX pharmacokinetics using a two-compartment NONMEM (Nonlinear mixed effects modeling) model has been similarly demonstrated in adults. It is possible that future studies are needed to evaluate other models in the pediatric population. These studies could mirror studies that have been performed in healthy adult patients, administering target-controlled infusions (TCI) of DEX to target concentrations of 1–8 ng/mL. Using this delivery method, DEX pharmacokinetics followed a three-compartment allometric model in adults, with weight as the only covariate [5]. Perhaps such a model would also best fit the neonate, infant, and pediatric population. The challenge of administering DEX with TCI in these populations will hamper a rapid answer.

The pharmacology and pharmacokinetics of DEX administered by different routes remains a topic of continued investigation in the pediatric population. Intranasal DEX seems to follow the same two-compartmental model as does the intravenous route in children. A small study of 18 children evaluated the pharmacokinetics of 1 and 2 mcg/kg intranasal administration. The time to reach minimum serum concentrations required to achieve sedation (100 ng/mL) was 20 min and 10 min, respectively, with the doubling of dosage. Peak plasma concentrations of both doses were achieved at 47 min with 84% bioavailability, similar to previously reported studies in adults [6,7,8]. Similar outcomes were reported in an open-label study which administered 2–3 mcg/kg IN (Intra Nasal) DEX to healthy children (5 months–11 years) as an anxiolytic prior to ambulatory magnetic resonance imaging (MRI). The maximal sedation effect was at a median time of 45 min with a median decrease in heart rate of 15% from baseline [9].

A pharmacokinetic-pharmacodynamic model in children, to predict mean arterial blood pressure (MAP), heart rate (HR), and clearance, was presented for healthy Mexican children age 2–18 years. Using a two-compartment model and using an allometric model standardized for 70 kg, a single dose of 0.7 mcg/kg DEX was shown to follow a concentration–time relationship. A maximum mean arterial blood pressure reduction of 45% was predicted with an inhibitory concentration IC50 of 0.501 ng/mL, and a maximum heart rate reduction of 28.9% with an IC50 of 0.552 ng/mL. Clearance was lower in the pediatric population [10]. This predictability of serum concentrations of DEX to elicit a particular hemodynamic response (reduced heart rate and the biphasic effect on mean arterial pressure) is consistent with the adult literature. In healthy adult volunteers, however, NONMEM models generated with TCI DEX demonstrated that these parameters could be predicted from plasma concentration [11]. Future studies will be important in order to determine whether this relationship can also be predicted for the pediatric population.

The pharmacokinetics of DEX in children with compromised hepatic function must be considered. A single-center, open-label study of 0.5 mg/kg DEX followed by a 0.5 mcg/kg/h continuous DEX infusion in children 1 month–18 years of age demonstrated that DEX clearance is not affected by body weight in this population. Rather, clearance followed a two-compartment NONMEM model and was inversely proportional to the international normalized ratio (INR). Specifically, when the INR increased to 3.2, the DEX clearance decreased by 50%. It is important to recognize that in populations with compromised hepatic function, especially when involving shifts in INR, the clinician must consider modifying the DEX dosing to account for the decreased clearance [12].

The pharmacokinetics of DEX in the obese infant and pediatric population has not been studied. In obese adults, however, a two-compartment NONMEM model of DEX using lean body weight most accurately described DEX pharmacokinetics [13]. Consideration should be given to applying lean body weight when dosing obese children, particularly neonates who have an increased likelihood of decreased DEX clearance.

## 3. Updates on End-Organ Effects

### 3.1. Respiration and Airway Collapsibility

In contrast to traditional sedative agents, DEX appears to provide a neuropharmacological profile that seems to simulate natural sleep pathways when compared to other sedating agents [14,15,16,17,18]. This advantage, however, should not undermine the importance of maintaining respiratory monitoring during DEX administration.

DEX is unique in that its clinical effects on the airway mimic natural sleep. Recent literature compared critical closing airway pressures during natural sleep to that obtained during DEX sedation in children/adolescents with Down syndrome and persistent obstructive sleep apnea (OSA). These children with OSA were able to compensate for airway obstruction under DEX-induced sleep and demonstrated airway responses with DEX that paralleled those seen during natural sleep [19]. This ability of DEX to maintain spontaneous ventilation and upper airway tone makes it an attractive choice for drug-induced sleep endoscopy (DISE) and dynamic airway imaging, important studies needed when planning for surgical intervention [20,21,22,23]. DEX is considered to represent the gold standard for performing dynamic airway evaluations, particularly as it simulates natural conditions and preserves respiratory function [24].

Tracheal intubation without the use of muscle relaxants is commonly used in pediatric anesthesia and offers some important benefits. DEX has been shown to improve intubation conditions, increase tolerance to the presence of endotracheal tubes, decrease the required minimum alveolar concentration values of sevoflurane required to achieve smooth extubation, and diminish laryngeal responses to laryngeal and tracheal irritation [25,26,27,28,29]. DEX has also been shown to offer the important advantage of obtunding airway reflexes while maintaining stable hemodynamic and respiratory profiles in spontaneously ventilating children during rigid bronchoscopy [30].

A recent application of DEX is in its use for pulmonary function testing (PFT), a common diagnostic and assessment tool for patients with respiratory tract diseases. Administration of chloral hydrate, propofol, and midazolam can affect children’s expiratory flows when providing sedation for PFT. Recent literature supports the administration of intranasal DEX (2.64 mcg/kg) sedation for PFTs in children aged 1–3 years [31].

### 3.2. Inflammation and Immune System

**Figure 2:**
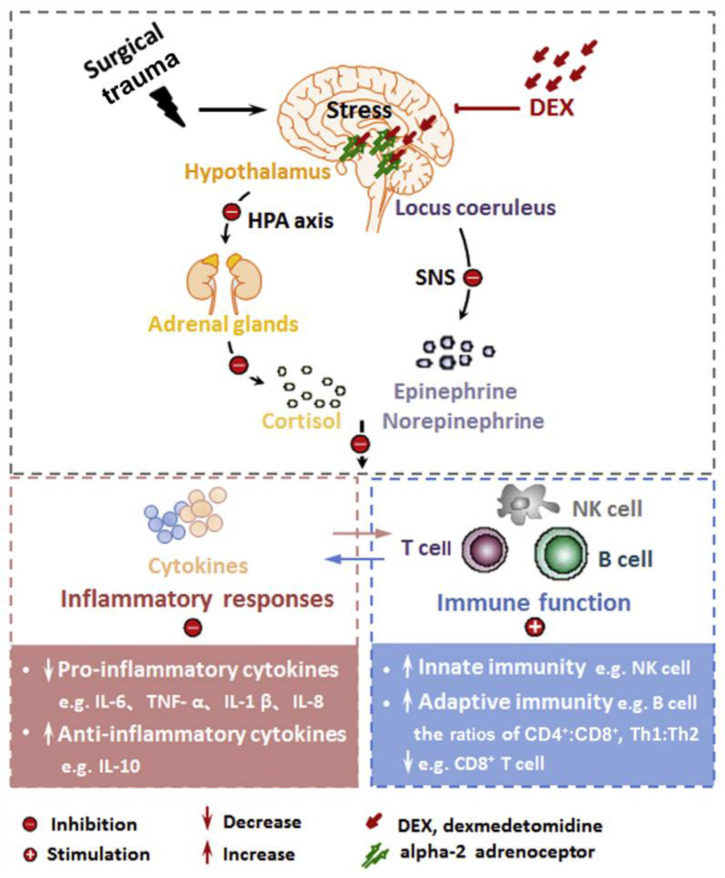
Schematic illustration that surgical trauma triggers perioperative stress, systemic inflammation, and immune suppression, all of which are negated by dexmedetomidine (DEX). HPA, hypothalamic-pituitary-adrenal; SNS, sympathetic nervous system [36].

Evidence continues to reveal that DEX possesses anti-inflammatory properties in adults and rodents [32,33]. Perioperative administration of DEX has been recently reported to suppress surgical stress and inflammation and preserve the immunity of surgical patients [34,35]. As an anesthesia adjuvant, it inhibits the concentrations of epinephrine, norepinephrine, cortisol, and blood glucose, and alleviates the perioperative stress in adults. (Figure 2) [36].

DEX has also been shown to inhibit the inflammatory response, stabilize the integrity of blood–spinal cord barrier [37], improve neuronal viability, and protect the spinal cord from ischemia– reperfusion injury by reducing microglial activation and inhibiting the toll-like receptor 4-mediated nuclear factor κB inflammatory system and caspase-3 dependent apoptosis [38]. In animal models, peri-neural administration of DEX attenuated inflammation in the sciatic nerve via reducing inflammatory cytokine levels [39]. Future studies will be important to determine whether the effects observed in adults and animals are replicated in the pediatric population.

### 3.3. Central Nervous System

**Figure 3:**
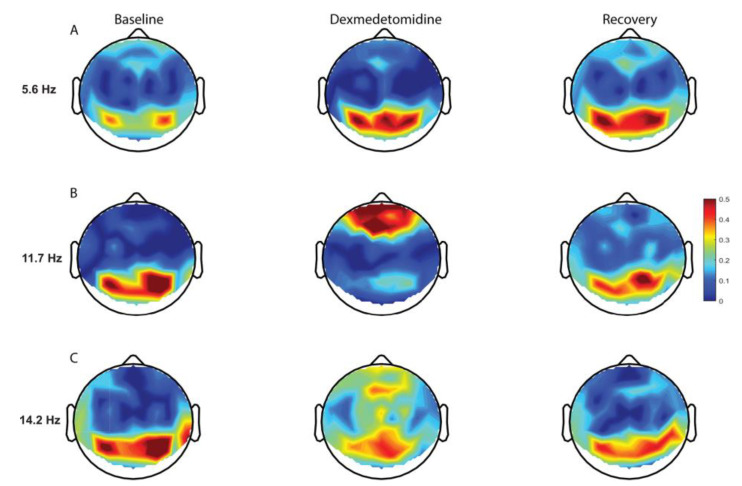
Topographic electroencephalogram maps detailing group-averaged global coherence for each electroencephalogram frequency of interest. (**A**) Dexmedetomidine is associated with increased occipital theta global coherence. (**B**) Dexmedetomidine is associated with a shift in the globally coherent occipital awake alpha to frontal regions. (**C**) Dexmedetomidine is associated with globally coherent fronto-central spindle oscillations [14].

DEX has been shown in the pediatric population to elicit electroencephalogram (EEG) activity similar to natural, non-REM (Rapid Eye Movement) sleep [40]. The specific effects of DEX at the cortical level have not yet been studied in children. It is important, though, to examine such data in adults, in order to guide future pediatric studies and clinical applications. In adults, electroencephalograms (EEGs) have been evaluated during DEX-induced sleep (1 mcg/kg DEX bolus followed by 0.7 mcg/kg/h continuous infusion). DEX was associated with increased slow-delta oscillations across the entire scalp, increased theta oscillations in occipital regions, increased spindle oscillations in frontal regions, and decreased beta oscillations across the entire scalp, all similar findings during natural sleep (Figure 3) [14].

**Figure 4:**
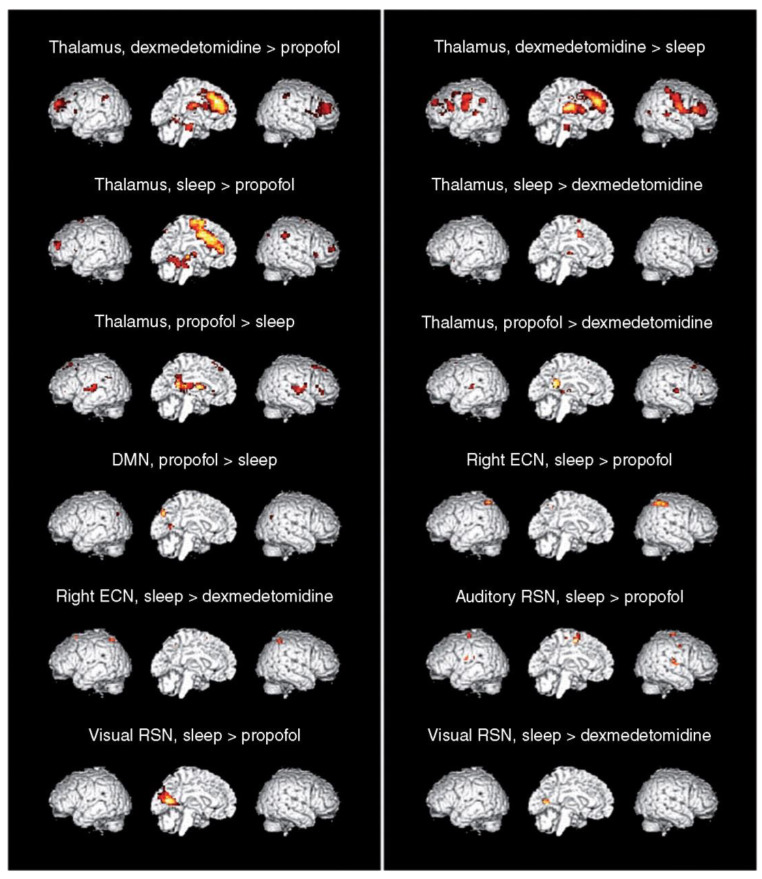
Contrast images showing regions where functional connectivity is higher during dexmedetomidine- than propofol-induced unresponsiveness (dexmedetomidine > propofol), dexmedetomidine than N3 sleep (dexmedetomidine > sleep), N3 sleep than propofol (sleep > propofol), N3 sleep than dexmedetomidine (sleep > dexmedetomidine), propofol than N3 sleep (propofol > sleep), or propofol than dexmedetomidine (propofol > dexmedetomidine). Only those contrasts where significant differences (false discovery rate-corrected *p* < 0.05) were found are shown, as was the case for connectivity with the thalamus (thalamus), within the default mode network (DMN), the right executive control network (right ECN), the auditory network (auditory resting state network RSN (Auditory resting state network)), and the visual network (visual RSN). Contrast images are superimposed on a canonical three-dimensional brain representation providing a left, right, and sagittal view of the brain. A left or right view of the internal face of the brain was chosen as a function of the presence of significant clusters or not. When no significant clusters were visible on one view, the other one was chosen [15].

A small randomized controlled trial of elderly (>65 years) non-intubated adults in the intensive care unit evaluated the sleep patterns during administration of 0.1 mcg/kg/h DEX for 15 h (initiated at 5 p.m.). They reported prolonged total sleep time, increased time in stage 2 non-rapid eye movement (REM) sleep (N2) (from 15.5% to 43.5%), increased sleep efficiency, and improved subjective sleep quality [41]. The target site of DEX-induced sedation at the cortical level has not been elucidated in the pediatric population. In healthy adults, however, functional MRI was used to evaluate and compare the neural correlates of natural non-REM N3 sleep with propofol- or DEX-induced sedation. Thalamic functional connectivity was reduced in both natural and drug-induced sleep states. However, N3 and DEX sedation had comparatively less effect at the medial prefrontal/anterior cingulate cortex and mesopontine areas than propofol. The better-preserved cortical networks with DEX could explain the faster recovery to oriented responsiveness following discontinuation of DEX as compared to propofol (Figure 4) [15].

DEX in adult volunteers (1 mcg/kg bolus then 0.7 mcg/kg/h) has been shown to reduce mean strength of cortical networks without impairing degree of distributions, while still modulating functional connectivity within and between all resting networks [42]. DEX sedation, unlike propofol, has been shown to partially preserve processing of words in adult volunteers [43]. Similar studies evaluating the EEGs across the specific cortical areas have to date not been specifically performed in the pediatric population. However, important adult studies may provide ideas for future pediatric investigations.

### 3.4. Cardiovascular System

A common concern with this novel sedative agent is bradycardia. Although there are no absolute contraindications to DEX, caution should be exercised in patients with depressed left ventricular function, recent high-degree AV (atrioventricular) block, and volume depletion, in children receiving digoxin, beta adrenergic blockers, calcium channel blockers, or other agents that predispose to bradycardia or hypotension. An earlier case report cites extreme bradycardia in an infant receiving DEX and digoxin [44]. A recent case report of two postoperative infants (cardiac surgery) reports cardiac arrest when co-administering DEX and amiodarone [45]. A case report in an adult describes a similar scenario. Two hours after initiating DEX to a patient being treated with amiodarone for tachycardia, cardiac arrest occurred. A review of the ECGs of the patient prior to the event demonstrated ominous atrioventricular abnormalities with DEX [46].

The pediatric literature reports that anticholinergics should be avoided as a treatment for DEX-induced bradycardia in the absence of hemodynamic instability. Small doses of glycopyrrolate can elicit extreme hypertension when administered to children sedated and receiving DEX [47]. Even when administered to children as a pretreatment to avoid DEX-induced bradycardia, a retrospective review reports similar responses: The systolic blood pressure increased by a percentage increase of up to 36% compared to the group that did not receive anticholinergics [48].

The DEX-induced bradycardia has, to date, not been reported to be of clinical relevance or require pharmacologic treatment. In adults, the drop in heart rate during DEX sedation mimics the heart rate response observed during natural and DEX-induced sleep in adults: Natural and DEX-induced sleep demonstrated changes in heart rate that were not statistically different [49]. Further studies will be important to determine whether, similar to adults, the heart rate response to DEX in the pediatric population is similar to those observed during natural sleep.

In adults, a recent study examined the paroxysmal supraventricular tachycardia (PSVT) inducibility during electrophysiology studies and ablation when DEX was used in addition to fentanyl and midazolam. The authors concluded that the administration of DEX was not associated with a reduction in PSVT inducibility [50]. The positive effect of DEX was reported on two children with recurrent supraventricular tachycardia previously in need of cardioversion, which spontaneously returned to sinus rhythm within 20–40 min after intranasal administration of a 4 mcg/kg dose of DEX in repeated occasions [51].

### 3.5. Thermal Regulation

DEX decreases vasoconstriction and may have a role in altering thermoregulatory shivering [52]. The controversy about the effectiveness of DEX for the prevention of shivering is still ongoing, with different results reported in the literature. As there are no pediatric studies to date, the adult literature reveals important findings. A recent meta-analysis including 39 trials, with 2478 patients, showed that DEX may prevent the incidence of post-operative shivering and has superiority over placebo, but not over other anti-shivering agents [53]. Physicians should be aware of febrile episodes that have been reported during DEX administration. In recent adult case series, nine cardiovascular intensive care unit patients experienced hyperthermia (>38.5 °C) during DEX administration at a dose range of 0.8–1.3 μg/kg/h. The hyperthermia was resolved 3 h (1–8) hours after discontinuation of DEX [54]. Vigilance is recommended to temperature monitoring in adult and pediatric patients receiving DEX, until the relationship between DEX and hyperthermia is further examined.

## 4. Updates on Organ-Protective Effects

### 4.1. Neuroprotective Effects

The neurotoxicity of anesthetics on the immature nervous system has become and remains a common concern in the medical community. In recent years, the neuroprotective effect of DEX has been increasingly studied. There is substantial in vivo and in vitro evidence that DEX has neuroprotective properties [55]. The data in pediatrics is limited and examining the existence of associations between early anesthetic exposure of DEX and long-term neurocognitive function in pediatric population is required. The adult data as well as animal and bench studies are promising. Studies have shown that DEX can alleviate the brain damage induced by anesthetics in animals by reducing the apoptosis in several cortical and subcortical brain regions [56,57,58]. A recent animal study that examined the effect of DEX on cognitive decline showed that DEX reversed the damage-associated molecular pattern-induced cognitive decline and inflammation via both vagomimetic and humoral pathways [59]. Recent literature also showed that DEX protects the spinal cord against lidocaine-induced spinal neurotoxicity through activating the α2 adrenergic receptor, which subsequently stimulated protein kinase C and inhibited glutamate release [60].

Traumatic brain injury (TBI) has been shown to involve injuries including microvascular ischemia, autonomic dysregulation, and neuronal excitotoxicity. Currently, there is no FDA-approved pharmacological therapy available that would reduce cell death following TBI. In traumatic animal model, DEX not only prevented tissue loss and cell death, but also reduced axonal injury and synaptic degeneration caused by TBI, resulting in improvement of motor function [61]. Cerebral ischemia is associated with an increase in circulating and extracellular brain catecholamine concentrations. The treatment with agents that are capable of reducing the release of norepinephrine in the brain (e.g., alpha 2-agonists) may provide protection against the damaging effect of cerebral ischemia. The neuroprotective effects of DEX are mediated by its binding to I1-imidazoline receptor and modulating histone acetylation via phospho-extracellular signal-related kinase pathways [62,63]. In addition, DEX has been shown to preserve neurological function by increasing the phosphorylation of protein kinase B and cAMP (Cyclic adenosine monophosphate) Cyclic adenosine monophosphate response element-binding protein and subsequently upregulating the expression of the antiapoptotic factors [64]. In a mouse model of intracerebral hemorrhage, DEX improved neurological deficits and brain injury by inhibiting mitochondrial dysfunction-derived oxidative stress [65]. Various animal models with complete and incomplete as well as transient and permanent ischemic injury have demonstrated the neuroprotective effects of DEX in animal models of ischemia/reperfusion [66].

One of the most interesting directions of DEX research involves its potential for neuroprotection, particularly in children. To date, only DEX and xenon have been proposed to be neuroprotective in animal studies [67]. Particularly as there is a growing interest and public concern over the potential effect of anesthetics and sedatives on the developing neonatal brain, DEX as a potentially neuroprotective agent should be further examined [68,69,70,71].

### 4.2. Renoprotective Effects

Intraoperative DEX infusion has a possible protective effect on cardiac surgery-associated acute kidney injury [72,73,74,75]. Similar data were reported in a double-blind, randomized clinical trial in children (6 months–6 years) who received intravenous iodine contrast for cardiac angiography. The use of DEX as an adjuvant to sedative agents decreased elevation in plasma endothelin, renin, and markers of acute renal injury in these children [76]. Current evidence demonstrates that the underlying renoprotective mechanism is achieved by promoting renal blood flow via inhibiting vasoconstriction and promoting a diuresis effect via decreasing renin and arginine vasopressin and increasing glomerular filtration, reducing reactive oxygen species, decreasing systemic inflammatory response, and reducing renal cell death [77,78].

### 4.3. Cardioprotective Effects

The potential cardioprotective effect of DEX has been reported in animal experiments [79,80]. New evidence showed that the cardioprotective effect of DEX is mediated via the cholinergic anti-inflammatory pathway [81]. By blocking the sympathetic nervous system, DEX blunts hemodynamic responses to perioperative stress, properly controls heart rate, and optimizes blood flow in the coronary arteries. A recent meta-analysis showed that DEX is an efficacious cardioprotective drug in adults and children undergoing cardiac surgery [82]. Ketamine has also been shown to have anti-inflammatory properties. A retrospective observational study that examined the influence of ketamine-DEX-based anesthesia on the release of cardiac biomarkers was compared with that of sevoflurane/sufentanil-based anesthesia and concluded that DEX-ketamine combination can attenuate myocardial ischemia-reperfusion injury during cardiac surgery [83].

## 5. Updates on Alternative Routes of Administration

DEX administration has been described by intramuscular, oral, buccal, and, most recently, by subcutaneous route [84]. The intranasal (IN) route is the most used extravascular route of administration of DEX in children. There is an increasing number of published reports describing the use of this route for sedation and premedication in the pediatric population: A recent meta-analysis of randomized, controlled trials comparing IN DEX with other IN or oral premedications revealed that IN DEX was non-irritating on administration and provided more satisfactory sedation at time of parent separation. It also reduced the need for rescue analgesics and post-operative nausea and vomiting [85].

Recent trials showed that 2–4 mcg/kg of IN DEX in the pediatric population produced successful sedation in 70% to 100%. The characteristics of these trials are summarized in Table 1 [31,86,87,88,89,90]. When compared to traditional agents used for pediatric sedation (i.e., midazolam, propofol, chloral hydrate), =IN DEX (1 to 3 mcg/kg) was effective in providing adequate procedural sedation without significant adverse events. A summary of the characteristics of these trials is included in Table 2 [91,92,93,94,95]. Although DEX has been shown to provide effective sedation for non-invasive procedures, there is an increasing number of recent studies describing the use of DEX in addition to the current available sedative agents (Table 3) [88,96,97].

The bioavailability of DEX by the oral route is very poor (16%) and administration by such a route is unwarranted [98].

Buccal DEX with or without midazolam has been recently examined in children requiring sedation for MRI studies. Of the 220 sedation encounters, 179 (81.4%) patients had satisfactory sedation (failure rate of almost 20%) with buccal DEX with or without oral midazolam [99]. The buccal route of administration can be challenging in the pediatric population. Perhaps future studies will demonstrate effective ways to ensure buccal dosing while, importantly, avoiding oral consumption.

## 6. Updates of Synergistic Effects

### 6.1. Regional Anesthesia

Approved only for intravenous administration, DEX utilization for regional blockade is an off-label usage that has been rarely described in the pediatric literature. Exploring its use in the adult population, with some extrapolation and comparison to published pediatric literature, however, can be valuable when considering DEX for pediatric regional practice. Specifically for lower extremity analgesia, a randomized controlled trial (RCT) examined the effect of 1 mcg/kg DEX added to 0.3% ropivacaine administered by caudal route for hemorrhoidectomy in adults. DEX had faster onset and longer duration of sensory block and analgesia. There were no clinically significant hemodynamic changes noted [100]. A meta-analysis of 10 RCTs evaluating DEX combined with bupivacaine for caudal anesthesia in the pediatric population reported similar results with longer post-operative analgesia and few rescue analgesics [101].

A prospective, randomized, double-blind study of healthy (ASA (American Society of Anesthesiologists) 1 and 2) children (1.5–18 years) compared 0.197% ropivacaine plain to that with 0.3 mcg/kg added DEX following inguinal hernia repair. The DEX group had less post-operative analgesic requirements and exhibited a lower incidence of emergence delirium [102].

DEX has also been similarly shown to decrease post-op narcotic requirements, increase the time until first demand for post-op narcotics, accelerate onset of block, and extend duration of the block when used with 0.5% bupivacaine for femoral nerve blocks in adults. The addition of 75 mcg DEX had best results, albeit a higher risk of hypotension [103]. The dosing of DEX is important, as studies comparing 0.5% ropivacaine with and without 100 mcg DEX in adults for saphenous nerve block demonstrated only a 2-h advantage in duration of sensory block with DEX compared to control [104].

A meta-analysis of DEX as an adjuvant for brachial plexus blocks in adults evaluated 32 studies and suggested that DEX prolongs and expedites sensory and motor blockade. It prolongs analgesia, has increased patient satisfaction, and decreases post-operative oral opiate consumption. A 50–60 mcg dose in adults was found to decrease the risk of bradycardia and hypotension while providing the benefits of prolonged analgesia and blockade [105]. A randomized double-blind adult trial evaluated different dosages (1–2 mcg/kg) of DEX combined with 0.5% ropivacaine and compared to controls for interscalene brachial plexus blocks in adults. The duration of analgesia and sensory and motor blocks was longer in the DEX groups, with longest duration of analgesia in the group that received the higher dosage, albeit there was a higher incidence of hypotension in this group [106]. One mcg/kg added to a supraclavicular brachial plexus block with 0.75% ropivacaine and 2% lidocaine in adults demonstrated increased duration of motor and sensory blockade and decreased opiate demand for 24 h post-operatively [107].

Particularly as there are continued efforts and studies designed to minimize and reduce the anesthetic exposure to neonates and young children, the potential role of DEX as a valuable adjuvant to prolonging a regional anesthetic and decreasing the anesthetic and analgesic requirements holds significant promise.

### 6.2. Anesthesia

DEX has been administered via various routes as an adjuvant to pediatric anesthesia and sedation. Its role as a synergist, particularly when administered with intravenous anesthetics, has not yet been determined in the pediatric population. In the adult population, however, DEX has been shown to be synergistic with propofol: IV DEX administered as an adjunct to propofol anesthesia administered via a closed loop anesthesia delivery system decreased the propofol required for induction and maintenance by 15%. Hypotension and bradycardia were observed at higher frequency in those who received DEX (1 mcg/kg bolus then 0.5 mcg/kg/h.) [108]. Similar results have been shown with DEX administered by other routes. In adults, IN DEX in doses of 1 and 2 mcg/kg decreases propofol and remifentanil requirements when administered via TCI to a BIS (bispectral index) of 45–55 and NIBP (non-invasive blood pressure) within 20% of baseline. Two mcg/kg IN DEX had better effect on decreasing anesthesia requirements and 1 mcg/kg via the IV route had better anesthesia-sparing effects as compared to the same dose IN [109]. Similar results have been found in the pediatric population. A retrospective chart review of children (1 month–20 years) who received 0.5 mcg/kg DEX prior to a propofol anesthetic for MRI reported that the overall dose of propofol and incidence of hypotension was less in the DEX group as compared to those who received propofol alone [110].

In children, IV DEX premedication has been shown to decrease the minimum alveolar concentration (MAC) of sevoflurane required for a smooth extubation (MAC-EX) after tonsillectomy. Healthy children (3–7 years) who received 1 or 2 mcg/kg DEX prior to anesthesia induction had a dose-dependent decrease in MAC-EX compared to the saline controls (0.51, 0.83, and 1.40%, respectively). The EC95 (effective concentration) of sevoflurane also showed a dose-dependent decrease in those who received 2, 1, and 0 mcg/kg DEX (0.83, 1.07, and 1.73%, respectively) [111].

## 7. Post-Procedural Applications

### 7.1. Improving Post-Anesthesia Recovery

DEX has been shown to offer benefits for weaning pediatric patients following rigid bronchoscopy. Critically ill children who were unable to be extubated following rigid bronchoscopy were maintained on either DEX (1 mcg/kg bolus followed by 0.8 mcg/kg/h continuous infusion) or a remifentanil (6–10 mcg/kg/h) and propofol (1–3 mg/kg/h) infusion. The DEX group achieved spontaneous ventilation sooner and had a higher rate of first-attempt extubation success (96.7 vs. 77.8%) [112].

Although not described in the pediatric population, intratracheal DEX (1 mcg/kg) has been used with success to decrease the coughing on extubation in the adult population (18–60 years). Both the intratracheal and IV route for 1 mcg/kg DEX were shown to be equally effective and superior to controls who received IV saline [113].

DEX has been shown to be effective in decreasing incidence of post-anesthesia emergence delirium (ED) in the high-risk pediatric populations [114]. A prospective, single-center, double-blind, randomized study of healthy (ASA 1,2) children (3–14 years age) undergoing tonsillectomy demonstrated that 1 mcg/kg IV DEX decreased the incidence of ED by approximately 30% for up to 30 min post-operatively, without affecting the time to extubation [115]. The dosing required to decrease the incidence of ED may differ based on the inhalation anesthetic chosen. The 95% effective dose (ED95) of DEX for preventing ED following a tonsillectomy with desflurane was shown to be 0.38 mcg/kg [115]. A large, prospective, double-blind, randomized trial of children (4–10 years) undergoing tonsillectomy demonstrated that 0.5 mcg/kg rapid IV administration of DEX 5 min prior to termination of surgery decreased the incidence of ED without clinically significant hemodynamic effects. As compared to the control group that received saline, the DEX group had a decreased opiate requirement in the post-anesthesia care period [116]. DEX via the oral route does not have optimal bioavailability. However, at doses of 1 mcg/kg, oral DEX has been shown to be an effective premedication to elicit effective sedation for parental separation and mask acceptance for dental rehabilitation in healthy children (2–6 years). The failure to demonstrate a difference in ED may be due to the low bioavailability and the inability to achieve adequate plasma serum levels to have a demonstrable effect [117].

The effect of DEX on ketamine-induced agitation and delirium in the pediatric population has, to date, not been studied. In the healthy adult population, 0.5 mcg/kg DEX added to 2 mg/kg IV ketamine decreased the incidence of delirium, reduced hemodynamic variability, and decreased post-procedure pain [118].

A prospective, double-blind, parallel group study similarly evaluated effect of DEX on the incidence of ED following elective day surgery in healthy (ASA 1,2) children (3–7 years). The 1 mcg/kg DEX was administered via the nasal route and compared to 4 mg/kg IN clonidine, both administered 45 min prior to induction of anesthesia. DEX was superior at decreasing the incidence and severity of ED and had a greater effect on decreasing the post-operative fentanyl requirements [95].

DEX may have other beneficial effects on recovery. These effects have not been specifically studied in children. However, in a randomized, double-blind study of adult patients at high risk of post-op nausea and vomiting, 0.5 mcg/kg IV DEX administered 30 min prior to completion of surgery and followed by DEX in an IV fentanyl-ketorolac patient-controlled analgesia (PCA) system decreased the incidence and severity of nausea for the first 3 h post-operatively and lowered the incidence of moderate and severe nausea for up to 48 h [119]. Directed studies examining the potential anti-emetic prophylactic role of DEX in the pediatric population still needs to be conducted.

### 7.2. Neonatal Intensive Care

There is limited literature on the use of DEX in the neonatal intensive care unit (NICU). A multi-centered pharmacokinetic study of intubated preterm and term neonates with up to 0.2 mcg/kg loading dose and 0.2 mcg/kg/h reported successful sedation conditions in up to 90% with no serious reported adverse events during the 6–24-h administration. Because of the longer elimination half-life and clearance in the premature, the authors recommended lowering the DEX dosing in this population [120].

Similarly, when comparing dosing recommendations up to 2 years of age, pharmacokinetic studies recommend decreasing DEX dosing in the neonates [2]. Recommended dosing regimens have included 0.2–0.3 mc/kg loading dose and continuous infusions of 0.2–0.2 mcg/kg/h titrated up to a median of 0.4 mcg/kg/h for 45–219 h [121]. Doses of 0.4–0.8 mcg/kg/h for up to 82 h have been described for infants (mean 0.7 months), with 56% reporting hemodynamic shifts at higher doses, not requiring suspension of therapy [122].

In up to 66% of those neonates receiving DEX infusions, opioid infusions have been able to be decreased within 24 h of initiating DEX [123]. A reduction in opiate as well as benzodiazepine requirements has similarly been described in a small group of 14 neonates (average 51 weeks post-gestation). Post-tracheotomy, the group that received DEX infusions demonstrated a significant reduced need for opiate and benzodiazepine infusions from an average of 24 days to less than 7 days and from an average of 21 days to 8.9 days, respectively [124].

There is literature to suggest that neonates can exhibit symptoms of withdrawal. A retrospective review of 38 infants who received an average of 11.1 days of DEX infusion in doses which exceeded 0.5 mcg/kg/h reported that 71% exhibited signs of withdrawal. Clonidine was used in one-third of those weaned [123].

### 7.3. Pediatric Intensive Care Unit

Despite a paucity of large studies examining DEX utilization in the pediatric intensive care unit (PICU) setting, it has become an increasing commonplace adjunct worldwide. A 2017 multinational European study (16 hospitals, four EU countries) reported that up to 6% of children in the PICU receive DEX [125]. DEX has been shown to have particular benefits when used as the primary sedative for less critically ill PICU children, allowing rapid achievement of targeted sedation levels and still maintaining ability for clinical assessments. It has also been shown to hasten weaning off the ventilator [126]. DEX has also been demonstrated to be effective for children (median age 16 months) who require sedation for non-invasive ventilation (NIV), in efforts to provide adequate sedation to those with underlying pulmonary conditions and avoid endotracheal intubation. In one study, 90% of children were successfully sedated and able to tolerate NIV [127]. Future studies are needed to determine whether DEX can play a pivotal role in children requiring NIV, facilitating ventilator synchronization, improving lung recruitment, and avoiding the need for an invasive ventilatory support.

In the PICU setting, DEX has been shown to be effective as a prophylactic treatment for junctional ectopic tachycardia (JET) in post-cardiac surgery. A metanalysis of seven randomized controlled trials representing 1616 patients reported a significant reduction in JET, ICU stay, and duration of mechanical ventilation in those who received DEX [128]. Caution should be considered in the long-term use of DEX infusions, as there is a case report which describes supraventricular tachycardia during DEX weaning of a 4-year-old without pre-existing cardiac disease [129].

### 7.4. Palliative Care

There has been interest in using DEX to provide sedation, anxiolysis, and analgesia for pediatric patients with severe symptoms near the end of life, while promoting arousable and interactive sedation compared to patients sedated with traditional sedative agents. Continuous intravenous infusion of DEX in the setting of end of life care has been reported in a series of nine pediatric patients (6 months–17 years), none of whom exhibited significant hemodynamic sequela [130]. Infusions up to a maximum of 12 days were associated with a statistically significant decrease in pain scores and a downward trend in opioid use. Recently, The Italian Drug Agency passed a landmark decision, approving DEX via IN and intravenous route for the palliative care of children outside of the ICU setting (in hospital or at home) and unresponsive to conventional therapy [131,132].

IN DEX in doses of 2–3 mcg/kg have been effective for sedating a child with epidermolysis bullosa and one with refractory dystonia, without significant adverse events or tachyphylaxis when used at home for up to three months [132,133].

Despite the limited studies on DEX in the pediatric palliative care setting, the recent Italian initiative and the optimistic reports are encouraging and deserve further study, particularly for end-of-life care and intractable sleep disorders.

## 8. Limitations of the Literature and Future Directions

A significant limitation in the interpretation of DEX literature in the pediatric population is the presence of numerous retrospective observational studies. Albeit, the numbers of patients are large, the retrospective nature and subjective data collection methods between providers challenge the validity of the results. Retrospective data collection rarely uses clearly defined outcome metrics. The advantage of DEX in certain pediatric populations deserves future study. Preferred routes of administration should be examined, specific for different procedures and settings. For example, in the autistic population, DEX (intranasal, intramuscular, intravenous) has demonstrated success for sedation for radiological and neurological (ABR (auditory brainstem response), EEG) procedures as well as for anxiolysis and as an adjuvant for minor emergent procedures [40,134,135,136,137,138,139].

In recent years, the potential organ-protective effect of DEX has become a major direction of research efforts. Currently the most important clinical and research problem in the field of pediatric anesthesia is anesthetic neurotoxicity. There is substantial in vivo and in vitro evidence that DEX has neuroprotective properties. Examining the existence of associations between early anesthetic exposure of DEX and long-term neurocognitive function in human is definitely required to confirm its neuroprotective beneficial effects.

## Figures and Tables

**Table 1:** Characteristics of dose-finding studies in specific settings.

Author	Year	Dose (ED = Effective Dose)		No. of Patients		Age	Procedure/Study	Onset Time (Median Time)		Sedation’s Success		Adverse Events
Li et al. [31]	2020	2.64 µg/kg (2.49–2.87 µg/kg ED 95%)		68		1–3 years	Pulmonary function testing	15.0 (12.3–19.0) min		91%		None
Tug et al. [86]	2015	3 µg/kg	4 µg/kg	30	30	1–10 years	MRI ^I^	31 ± 13 min	30 ± 9 min	30%	70%	None
Liu et al. [87]	2016	2 µg/kg	4 µg/kg	121		6 months –5 years	Preoperative sedation	15 min	25 min	85.5%	77.6%	One child in the 2 µg/kg group had a reduction in systolic blood pressure to 29%.
Yang et al. [88]	2019	3.3 (2.48–3.53 ED50) µg/kg and 3.7 (3.44–3.73 ED 95) µg/kg in the cyanotic group and 1.7 (1.00–2.03 ED50) µg/kg and 2.2 (1.96–2.23 ED95) µg/kg in the acyanotic group.		50 patients with congenital heart disease (23 in the cyanotic group and 27 in the acyanotic group)		≤18 months	Transthoracic echocardiography in children with congenital heart disease	13.9 min in the cyanotic group and 17.5 min in the acyanotic group		50%		None
Miller et al. [89]	2016	2.5–3 µg/kg		63		17.5 mean age	Transthoracic echocardiography	28 min		90%		Five patients with mild to moderate hypotension without need for intervention.
Tenney et al. [90]	2019	2 µg/kg		26		5.5–20.5 years	Magnetoencephalography in patients with medically intractable epilepsy	25.5 min		100%		None

^I^, Magnetic resonance imaging = MRI.

**Table 2:** Characteristics of the studies evaluating intranasal dexmedetomidine versus current available sedative agents.

Author	Year	Intervention	Dose	No. of Patients	Age	Procedure/ Intervention	Sedation’s Success	Adverse Events
Behrle et al. [91]	2017	IN Dex ^II^ vs. intravenous sedative medications	3 µg/kg Dex vs. Midazolam, Propofol, Pentobarbital, Ketamine	109 vs. 690	6 months–18 years	Non-invasive procedures	92% (with 39% receiving in Midazolam for sedation ≥ 45 min)	Not significant
Li et al. [92]	2020	IN Dex ^II^ vs. Oral Chloral Hydrate	1–3 µg/kg Dex	720	≤4 years	Ophthalmic examination, transthoracic echocardiograph, auditory brainstem response testing, CT ^III^ and MRI ^IV^ imaging	Higher success rate in the first group (RR 1.12)	Lower incidence of nausea and vomiting in the IN Dex ^II^ group
			25 to 80 mg/kg Chloral Idrate					
Zhang et al. [93]	2015	Two rescue doses of IN Dex ^II^ vs. second oral dose of Chloral Hydrate after 50 mg of oral Chloral Hydrate	1 and 2 µg/kg Dex	150	1–6 months	MRI ^IV^	94% and 98% in the IN Dex groups	None
			25 mg/kg Chloral Idrate					
Ghai et al. [94]	2017	IN Dex ^II^ vs. Oral Midazolam	2.5 µg/kg Dex	59	6 years	IV cannulation CT ^III^ scan	67% vs. 24% achieved a Ramsey Sedation Score higher than 4	None
			0.5 mg/kg Midazolam					
Mukherjee et al. [95]	2015	IN Dex ^II^ vs. IN ^I^ Clonidine before Sevoflurane anesthesia	1 µg/kg Dex	80	3–7 years	Preoperative sedation	Emergence agitation was lower in the first group	Nausea and vomiting (no statistical differences between the 2 cohorts)
			4 µg/kg Clonidine					

^I^, Intranasal = IN, ^II^, Intranasal dexmedetomidine = IN DEX, ^III^, Computed tomography = CT, ^IV^, Magnetic resonance imaging = MRI.

**Table 3:** Characteristics of the studies evaluating intranasal dexmedetomidine in association with available sedative agents.

Author	Year	Intervention	Dose	No. of Patients	Age	Procedure/Intervention	Onset Time	Sedation’s Success	Adverse Events
Cozzi et al. [96]	2017	IN Dex ^II^ + Oral Midazolam	3 µg/kg Dex	108	4 months–17 years	MRI	33 (10–65) min	84% (90% < 2 years, 94% < 1 year)	Not significant ^i^
			0.5 mg/kg Midazolam						
Bua et al. [97]	2018	IN Dex ^II^ + IN ^I^ Midazolam in case of failure	3 µg/kg Dex	52	Ex pre-term babes at 40 weeks of gestational age	Brain MRI	10 (IQR 8–12) min	51% with Dex only, 100% with Dex+ Midazolam	5% had apnea needing positive pressure ventilation 13% had brief self-resolving desaturation (SpO_2_ < 94%)
			0.2 mg/kg Midazolam						
Yang et al. [88]	2019	IN Dex ^II^ + IN ^I^ Ketamine	2 µg/kg Dex	17948	21 (IQR 10–34) months	Color doppler ultrasound, Pulmonary function, EEG ^III^, MRI ^IV^, ECG^V^, auditory brainstem response testing, Fundus examination, CT ^VI^	15 (15–20) min	93% (1.8% required intranasal sedation rescue) ^ii^	0.02% emergent airway intervention 0.01% cardiac arrhythmias 0.59% minor adverse Events ^iii^
			1 mg/kg Ketamine						

^I^, Intranasal = IN; ^II^, Intranasal dexmedetomidine = IN DEX; ^III^, Electroencephalography = EEG; ^IV^, Magnetic resonance imaging = MRI; ^V^ Electrocardiography = ECG; ^VI^, Computed tomography = CT. ^i^ 5% experienced transient oxygen desaturation without need of ventilation, 3% experienced transient self-limiting hypotension, 8% had transient self-limiting bradycardia, 2% had vomiting. ^ii^ Intranasal sedation rescue was defined as sedation success using intranasal additional bolus dose of 1 μg/kg dexmedetomidine and 0.5 mg/kg of ketamine. ^iii^ 0.3% post-operative nausea and vomiting, 0.22% SpO_2_ reduction < 90%, 0.11% upper airway obstruction, 0.06% delayed awakening, 0.02% unexpected changes in heart rate or blood pressure > 20% normal, age-adjusted values and given pharmacological intervention, 0.02% rash.

## References

[1] Mahmoud M., Mason K.P. (2015). Dexmedetomidine: Review, update, and future considerations of paediatric perioperative and periprocedural applications and limitations. Br. J. Anaesth..

[2] Su F., Gastonguay M.R., Nicolson S.C., DiLiberto M., Ocampo-Pelland A., Zuppa A.F. (2016). Dexmedetomidine Pharmacology in Neonates and Infants After Open Heart Surgery. Anesth. Analg..

[3] Greenberg R.G., Wu H., Laughon M., Capparelli E., Rowe S., Zimmerman K.O., Smith P.B., Cohen-Wolkowiez M. (2017). Population pharmacokinetics of dexmedetomidine in infants. J. Clin. Pharmacol..

[4] van Dijkman S.C., De Cock P., Smets K., Decaluwe W., Smits A., Allegaert K., Vande Walle J., De Paepe P., Della Pasqua O. (2019). Dose rationale and pharmacokinetics of dexmedetomidine in mechanically ventilated new-borns: Impact of design optimisation. Eur. J. Clin. Pharmacol..

[5] Hannivoort L.N., Eleveld D.J., Proost J.H., Reyntjens K.M., Absalom A.R., Vereecke H.E., Struys M.M. (2015). Development of an Optimized Pharmacokinetic Model of Dexmedetomidine Using Target-controlled Infusion in Healthy Volunteers. Anesthesiology.

[6] Miller J.W., Balyan R., Dong M., Mahmoud M., Lam J.E., Pratap J.N., Paquin J.R., Li B.L., Spaeth J.P., Vinks A. (2018). Does intranasal dexmedetomidine provide adequate plasma concentrations for sedation in children: A pharmacokinetic study. Br. J. Anaesth..

[7] Anttila M., Penttilä J., Helminen A., Vuorilehto L., Scheinin H. (2003). Bioavailability of dexmedetomidine after extravascular doses in healthy subjects. Br. J. Clin. Pharmacol..

[8] Iirola T., Vilo S., Manner T., Aantaa R., Lahtinen M., Scheinin M., Olkkola K.T. (2011). Bioavailability of dexmedetomidine after intranasal administration. Eur. J. Clin. Pharmacol..

[9] Uusalo P., Guillaume S., Siren S., Manner T., Vilo S., Scheinin M., Saari T.I. (2020). Pharmacokinetics and Sedative Effects of Intranasal Dexmedetomidine in Ambulatory Pediatric Patients. Anesth. Analg..

[10] Perez-Guille M.G., Toledo-Lopez A., Rivera-Espinosa L., Alemon-Medina R., Murata C., Lares-Asseff I., Chavez-Pacheco J.L., Gomez-Garduno J., Zamora Gutierrez A.L., Orozco-Galicia C. (2018). Population Pharmacokinetics and Pharmacodynamics of Dexmedetomidine in Children Undergoing Ambulatory Surgery. Anesth. Analg..

[11] Colin P.J., Hannivoort L.N., Eleveld D.J., Reyntjens K., Absalom A.R., Vereecke H.E.M., Struys M. (2017). Dexmedetomidine pharmacodynamics in healthy volunteers: 2. Haemodynamic profile. Br. J. Anaesth..

[12] Damian M.A., Hammer G.B., Elkomy M.H., Frymoyer A., Drover D.R., Su F. (2020). Pharmacokinetics of Dexmedetomidine in Infants and Children After Orthotopic Liver Transplantation. Anesth. Analg..

[13] Rolle A., Paredes S., Cortinez L.I., Anderson B.J., Quezada N., Solari S., Allende F., Torres J., Cabrera D., Contreras V. (2018). Dexmedetomidine metabolic clearance is not affected by fat mass in obese patients. Br. J. Anaesth..

[14] Akeju O., Kim S.E., Vazquez R., Rhee J., Pavone K.J., Hobbs L.E., Purdon P.L., Brown E.N. (2016). Spatiotemporal Dynamics of Dexmedetomidine-Induced Electroencephalogram Oscillations. PLoS ONE.

[15] Guldenmund P., Vanhaudenhuyse A., Sanders R.D., Sleigh J., Bruno M.A., Demertzi A., Bahri M.A., Jaquet O., Sanfilippo J., Baquero K. (2017). Brain functional connectivity differentiates dexmedetomidine from propofol and natural sleep. Br. J. Anaesth..

[16] Nelson L.E., Lu J., Guo T., Saper C.B., Franks N.P., Maze M. (2003). The alpha2-adrenoceptor agonist dexmedetomidine converges on an endogenous sleep-promoting pathway to exert its sedative effects. Anesthesiology.

[17] Doze V.A., Chen B.X., Maze M. (1989). Dexmedetomidine produces a hypnotic-anesthetic action in rats via activation of central alpha-2 adrenoceptors. Anesthesiology.

[18] Hsu Y.W., Cortinez L.I., Robertson K.M., Keifer J.C., Sum-Ping S.T., Moretti E.W., Young C.C., Wright D.R., Macleod D.B., Somma J. (2004). Dexmedetomidine pharmacodynamics: Part I: Crossover comparison of the respiratory effects of dexmedetomidine and remifentanil in healthy volunteers. Anesthesiology.

[19] Mahmoud M., Ishman S.L., McConnell K., Fleck R., Shott S., Mylavarapu G., Gutmark E., Zou Y., Szczesniak R., Amin R.S. (2017). Upper Airway Reflexes are Preserved During Dexmedetomidine Sedation in Children With Down Syndrome and Obstructive Sleep Apnea. J. Clin. Sleep Med..

[20] Chatterjee D., Friedman N., Shott S., Mahmoud M. (2014). Anesthetic dilemmas for dynamic evaluation of the pediatric upper airway. Semin. Cardiothorac. Vasc. Anesth..

[21] Mahmoud M., Gunter J., Donnelly L.F., Wang Y., Nick T.G., Sadhasivam S. (2009). A comparison of dexmedetomidine with propofol for magnetic resonance imaging sleep studies in children. Anesth. Analg..

[22] Mahmoud M., Jung D., Salisbury S., McAuliffe J., Gunter J., Patio M., Donnelly L.F., Fleck R. (2013). Effect of increasing depth of dexmedetomidine and propofol anesthesia on upper airway morphology in children and adolescents with obstructive sleep apnea. J. Clin. Anesth..

[23] Padiyara T.V., Bansal S., Jain D., Arora S., Gandhi K. (2020). Dexmedetomidine versus propofol at different sedation depths during drug-induced sleep endoscopy: A randomized trial. Laryngoscope.

[24] Chang E.T., Certal V., Song S.A., Zaghi S., Carrasco-Llatas M., Torre C., Capasso R., Camacho M. (2017). Dexmedetomidine versus propofol during drug-induced sleep endoscopy and sedation: A systematic review. Sleep Breath. Schlaf Atm..

[25] Di M., Han Y., Yang Z., Liu H., Ye X., Lai H., Li J., ShangGuan W., Lian Q. (2017). Tracheal extubation in deeply anesthetized pediatric patients after tonsillectomy: A comparison of high-concentration sevoflurane alone and low-concentration sevoflurane in combination with dexmedetomidine pre-medication. BMC Anesthesiol..

[26] Fan Q., Hu C., Ye M., Shen X. (2015). Dexmedetomidine for tracheal extubation in deeply anesthetized adult patients after otologic surgery: A comparison with remifentanil. BMC Anesthesiol..

[27] Yao Y., Qian B., Lin Y., Wu W., Ye H., Chen Y. (2015). Intranasal dexmedetomidine premedication reduces minimum alveolar concentration of sevoflurane for laryngeal mask airway insertion and emergence delirium in children: A prospective, randomized, double-blind, placebo-controlled trial. Paediatr. Anaesth..

[28] Wei L., Deng X., Sui J., Wang L., Liu J. (2015). Dexmedetomidine Improves Intubating Conditions Without Muscle Relaxants in Children After Induction With Propofol and Remifentanil. Anesth. Analg..

[29] He L., Wang X., Zheng S. (2014). Effects of dexmedetomidine on sevoflurane requirement for 50% excellent tracheal intubation in children: A randomized, double-blind comparison. Paediatr. Anaesth..

[30] Chen K.Z., Ye M., Hu C.B., Shen X. (2014). Dexmedetomidine vs remifentanil intravenous anaesthesia and spontaneous ventilation for airway foreign body removal in children. Br. J. Anaesth..

[31] Li S., Liu H., Zhang J., Liu Y., Yu Q., Sun M., Tian Q., Yang F., Lei Y., Liu X. (2020). The 95% effective dose of intranasal dexmedetomidine sedation for pulmonary function testing in children aged 1–3 years: A biased coin design up-and-down sequential method. J. Clin. Anesth..

[32] Xu L., Bao H., Si Y., Wang X. (2013). Effects of dexmedetomidine on early and late cytokines during polymicrobial sepsis in mice. Inflamm. Res..

[33] Chen Y., Miao L., Yao Y., Wu W., Wu X., Gong C., Qiu L., Chen J. (2015). Dexmedetomidine Ameliorate CLP-Induced Rat Intestinal Injury via Inhibition of Inflammation. Mediat. Inflamm..

[34] Ferreira J.A., Bissell B.D. (2018). Misdirected Sympathy: The Role of Sympatholysis in Sepsis and Septic Shock. J. Intensive Care Med..

[35] Li Y., Wang B., Zhang L.L., He S.F., Hu X.W., Wong G.T., Zhang Y. (2016). Dexmedetomidine Combined with General Anesthesia Provides Similar Intraoperative Stress Response Reduction When Compared with a Combined General and Epidural Anesthetic Technique. Anesth. Analg..

[36] Wang K., Wu M., Xu J., Wu C., Zhang B., Wang G., Ma D. (2019). Effects of dexmedetomidine on perioperative stress, inflammation, and immune function: Systematic review and meta-analysis. Br. J. Anaesth..

[37] Liu J., Zhang S., Fan X., Yuan F., Dai J., Hu J. (2019). Dexmedetomidine Preconditioning Ameliorates Inflammation and Blood-Spinal Cord Barrier Damage After Spinal Cord Ischemia-Reperfusion Injury by Down-Regulation High Mobility Group Box 1-Toll-Like Receptor 4-Nuclear Factor kappaB Signaling Pathway. Spine.

[38] Sun Z., Zhao T., Lv S., Gao Y., Masters J., Weng H. (2018). Dexmedetomidine attenuates spinal cord ischemia-reperfusion injury through both anti-inflammation and anti-apoptosis mechanisms in rabbits. J. Transl. Med..

[39] Huang Y., Lu Y., Zhang L., Yan J., Jiang J., Jiang H. (2014). Perineural dexmedetomidine attenuates inflammation in rat sciatic nerve via the NF-kappaB pathway. Int. J. Mol. Sci..

[40] Mason K.P., O’Mahony E., Zurakowski D., Libenson M.H. (2009). Effects of dexmedetomidine sedation on the EEG in children. Paediatr. Anaesth..

[41] Wu X.H., Cui F., Zhang C., Meng Z.T., Wang D.X., Ma J., Wang G.F., Zhu S.N., Ma D. (2016). Low-dose Dexmedetomidine Improves Sleep Quality Pattern in Elderly Patients after Noncardiac Surgery in the Intensive Care Unit: A Pilot Randomized Controlled Trial. Anesthesiology.

[42] Hashmi J.A., Loggia M.L., Khan S., Gao L., Kim J., Napadow V., Brown E.N., Akeju O. (2017). Dexmedetomidine Disrupts the Local and Global Efficiencies of Large-scale Brain Networks. Anesthesiology.

[43] Kallionpaa R.E., Scheinin A., Kallionpaa R.A., Sandman N., Kallioinen M., Laitio R., Laitio T., Kaskinoro K., Kuusela T., Revonsuo A. (2018). Spoken words are processed during dexmedetomidine-induced unresponsiveness. Br. J. Anaesth..

[44] Berkenbosch J.W., Tobias J.D. (2003). Development of bradycardia during sedation with dexmedetomidine in an infant concurrently receiving digoxin. Pediatric Crit. Care Med..

[45] Fritock M.D., Ing R.J., Twite M.D. (2017). Cardiac Arrest in 2 Neonates Receiving Amiodarone and Dexmedetomidine. J. Cardiothorac. Vasc. Anesth..

[46] Ohmori T., Shiota N., Haramo A., Masuda T., Maruyama F., Wakabayashi K., Adachi Y.U., Nakazawa K. (2015). Post-operative cardiac arrest induced by co-administration of amiodarone and dexmedetomidine: A case report. J. Intensive Care.

[47] Mason K.P., Zgleszewski S., Forman R.E., Stark C., DiNardo J.A. (2009). An exaggerated hypertensive response to glycopyrrolate therapy for bradycardia associated with high-dose dexmedetomidine. Anesth. Analg..

[48] Subramanyam R., Cudilo E.M., Hossain M.M., McAuliffe J., Wu J., Patino M., Gunter J., Mahmoud M. (2015). To Pretreat or Not to Pretreat: Prophylactic Anticholinergic Administration Before Dexmedetomidine in Pediatric Imaging. Anesth. Analg..

[49] Kang D., Lim C., Shim D.J., Kim H., Kim J.W., Chung H.J., Shin Y., Kim J.D., Ryu S.J. (2019). The correlation of heart rate between natural sleep and dexmedetomidine sedation. Korean J. Anesthesiol..

[50] Slupe A.M., Minnier J., Raitt M.H., Zarraga I.G.E., MacMurdy K.S., Jessel P.M. (2019). Dexmedetomidine Sedation for Paroxysmal Supraventricular Tachycardia Ablation Is Not Associated with Alteration of Arrhythmia Inducibility. Anesth. Analg..

[51] Hultin M., Sundberg E. (2018). Spontaneous Conversions of Supraventricular Tachycardia to Sinus Rhythm in Children After Premedication with Intranasal Dexmedetomidine: A Case Report. A A Pract..

[52] Botros J.M., Mahmoud A.M.S., Ragab S.G., Ahmed M.A.A., Roushdy H.M.S., Yassin H.M., Bolus M.L., Goda A.S. (2018). Comparative study between Dexmedetomidine and Ondansteron for prevention of post spinal shivering. A randomized controlled trial. BMC Anesthesiol..

[53] Liu Z.X., Xu F.Y., Liang X., Zhou M., Wu L., Wu J.R., Xia J.H., Zou Z. (2015). Efficacy of dexmedetomidine on postoperative shivering: A meta-analysis of clinical trials. Can. J. Anaesth. J. Can. D’anesthésie.

[54] Kruger B.D., Kurmann J., Corti N., Spahn D.R., Bettex D., Rudiger A. (2017). Dexmedetomidine-Associated Hyperthermia: A Series of 9 Cases and a Review of the Literature. Anesth. Analg..

[55] Pan W., Lin L., Zhang N., Yuan F., Hua X., Wang Y., Mo L. (2016). Neuroprotective Effects of Dexmedetomidine Against Hypoxia-Induced Nervous System Injury are Related to Inhibition of NF-kappaB/COX-2 Pathways. Cell. Mol. Neurobiol..

[56] Perez-Zoghbi J.F., Zhu W., Grafe M.R., Brambrink A.M. (2017). Dexmedetomidine-mediated neuroprotection against sevoflurane-induced neurotoxicity extends to several brain regions in neonatal rats. Br. J. Anaesth..

[57] Shan Y., Yang F., Tang Z., Bi C., Sun S., Zhang Y., Liu H. (2018). Dexmedetomidine Ameliorates the Neurotoxicity of Sevoflurane on the Immature Brain Through the BMP/SMAD Signaling Pathway. Front. Neurosci..

[58] Wang X., Shan Y., Tang Z., Gao L., Liu H. (2019). Neuroprotective effects of dexmedetomidine against isoflurane-induced neuronal injury via glutamate regulation in neonatal rats. Drug Des. Dev. Ther..

[59] Hu J., Vacas S., Feng X., Lutrin D., Uchida Y., Lai I.K., Maze M. (2018). Dexmedetomidine Prevents Cognitive Decline by Enhancing Resolution of High Mobility Group Box 1 Protein-induced Inflammation through a Vagomimetic Action in Mice. Anesthesiology.

[60] Xu H., Zhao B., She Y., Song X. (2018). Dexmedetomidine ameliorates lidocaine-induced spinal neurotoxicity via inhibiting glutamate release and the PKC pathway. Neurotoxicology.

[61] Wu J., Vogel T., Gao X., Lin B., Kulwin C., Chen J. (2018). Neuroprotective effect of dexmedetomidine in a murine model of traumatic brain injury. Sci. Rep..

[62] Hu S.P., Zhao J.J., Wang W.X., Liu Y., Wu H.F., Chen C., Yu L., Gui J.B. (2017). Dexmedetomidine increases acetylation level of histone through ERK1/2 pathway in dopamine neuron. Hum. Exp. Toxicol..

[63] Wang Y., Han R., Zuo Z. (2016). Dexmedetomidine post-treatment induces neuroprotection via activation of extracellular signal-regulated kinase in rats with subarachnoid haemorrhage. Br. J. Anaesth..

[64] Bell M.T., Puskas F., Bennett D.T., Herson P.S., Quillinan N., Fullerton D.A., Reece T.B. (2014). Dexmedetomidine, an alpha-2a adrenergic agonist, promotes ischemic tolerance in a murine model of spinal cord ischemia-reperfusion. J. Thorac. Cardiovasc. Surg..

[65] Huang J., Jiang Q. (2019). Dexmedetomidine Protects Against Neurological Dysfunction in a Mouse Intracerebral Hemorrhage Model by Inhibiting Mitochondrial Dysfunction-Derived Oxidative Stress. J. Stroke Cerebrovasc. Dis..

[66] Cai Y., Xu H., Yan J., Zhang L., Lu Y. (2014). Molecular targets and mechanism of action of dexmedetomidine in treatment of ischemia/reperfusion injury. Mol. Med. Rep..

[67] Sabir H., Bishop S., Cohen N., Maes E., Liu X., Dingley J., Thoresen M. (2013). Neither xenon nor fentanyl induces neuroapoptosis in the newborn pig brain. Anesthesiology.

[68] McCann M.E., Soriano S.G. (2019). Does general anesthesia affect neurodevelopment in infants and children?. BMJ.

[69] Andropoulos D.B. (2018). Effect of Anesthesia on the Developing Brain: Infant and Fetus. Fetal Diagn..

[70] Andropoulos D.B., Greene M.F. (2017). Anesthesia and Developing Brains—Implications of the FDA Warning. N. Engl. J. Med..

[71] U.S. Food and Drug Administration FDA Drug Safety Communication: FDA Approves Label Changes for Use of General Anesthetic and Sedation Drugs in Young Children. https://www.fda.gov/drugs/drug-safety-and-availability/fda-drug-safety-communication-fda-approves-label-changes-use-general-anesthetic-and-sedation-drugs.

[72] Cho J.S., Shim J.K., Soh S., Kim M.K., Kwak Y.L. (2016). Perioperative dexmedetomidine reduces the incidence and severity of acute kidney injury following valvular heart surgery. Kidney Int..

[73] Jo Y.Y., Kim J.Y., Lee J.Y., Choi C.H., Chang Y.J., Kwak H.J. (2017). The effect of intraoperative dexmedetomidine on acute kidney injury after pediatric congenital heart surgery: A prospective randomized trial. Medicine.

[74] Kwiatkowski D.M., Axelrod D.M., Sutherland S.M., Tesoro T.M., Krawczeski C.D. (2016). Dexmedetomidine Is Associated With Lower Incidence of Acute Kidney Injury After Congenital Heart Surgery. Pediatr. Crit. Care Med..

[75] Shi R., Tie H.T. (2017). Dexmedetomidine as a promising prevention strategy for cardiac surgery-associated acute kidney injury: A meta-analysis. Crit. Care (Lond. Engl.).

[76] Bayram A., Ulgey A., Baykan A., Narin N., Narin F., Esmaoglu A., Boyaci A. (2014). The effects of dexmedetomidine on early stage renal functions in pediatric patients undergoing cardiac angiography using non-ionic contrast media: A double-blind, randomized clinical trial. Paediatr. Anaesth..

[77] Ji F., Li Z., Young J.N., Yeranossian A., Liu H. (2013). Post-bypass dexmedetomidine use and postoperative acute kidney injury in patients undergoing cardiac surgery with cardiopulmonary bypass. PLoS ONE.

[78] Ammar A.S., Mahmoud K.M., Kasemy Z.A., Helwa M.A. (2016). Cardiac and renal protective effects of dexmedetomidine in cardiac surgeries: A randomized controlled trial. Saudi J. Anaesth..

[79] Gao J.M., Meng X.W., Zhang J., Chen W.R., Xia F., Peng K., Ji F.H. (2017). Dexmedetomidine Protects Cardiomyocytes against Hypoxia/Reoxygenation Injury by Suppressing TLR4-MyD88-NF-kappaB Signaling. BioMed Res. Int..

[80] Zhang J.J., Peng K., Zhang J., Meng X.W., Ji F.H. (2017). Dexmedetomidine preconditioning may attenuate myocardial ischemia/reperfusion injury by down-regulating the HMGB1-TLR4-MyD88-NF-small ka, CyrillicB signaling pathway. PLoS ONE.

[81] Zhang J., Xia F., Zhao H., Peng K., Liu H., Meng X., Chen C., Ji F. (2019). Dexmedetomidine-induced cardioprotection is mediated by inhibition of high mobility group box-1 and the cholinergic anti-inflammatory pathway in myocardial ischemia-reperfusion injury. PLoS ONE.

[82] Gong Z., Ma L., Zhong Y.L., Li J., Lv J., Xie Y.B. (2017). Myocardial protective effects of dexmedetomidine in patients undergoing cardiac surgery: A meta-analysis and systematic review. Exp. Ther. Med..

[83] Ríha H., Kotulák T., Březina A., Hess L., Kramář P., Szárszoi O., Netuka I., Pirk J. (2012). Comparison of the effects of ketamine-dexmedetomidine and sevoflurane-sufentanil anesthesia on cardiac biomarkers after cardiac surgery: An observational study. Physiol. Res..

[84] Uusalo P., Al-Ramahi D., Tilli I., Aantaa R.A., Scheinin M., Saari T.I. (2018). Subcutaneously administered dexmedetomidine is efficiently absorbed and is associated with attenuated cardiovascular effects in healthy volunteers. Eur. J. Clin. Pharmacol..

[85] Jun J.H., Kim K.N., Kim J.Y., Song S.M. (2017). The effects of intranasal dexmedetomidine premedication in children: A systematic review and meta-analysis. Can. J. Anaesth. J. Can. D’anesthésie.

[86] Tug A., Hanci A., Turk H.S., Aybey F., Isil C.T., Sayin P., Oba S. (2015). Comparison of Two Different Intranasal Doses of Dexmedetomidine in Children for Magnetic Resonance Imaging Sedation. Paediatr. Drugs.

[87] Liu S.E., Hui T., Wong S., Irwin M.G., Yuen V., Wong G.L.S. (2016). Abstract PR251: A Comparison of Two Doses of Intranasal Dexmedetomidine for Sedative Premedication in Children. Anesth. Analg..

[88] Yang F., Liu Y., Yu Q., Li S., Zhang J., Sun M., Liu L., Lei Y., Tian Q., Liu H. (2019). Analysis of 17 948 pediatric patients undergoing procedural sedation with a combination of intranasal dexmedetomidine and ketamine. Paediatr. Anaesth..

[89] Miller J.W., Divanovic A.A., Hossain M.M., Mahmoud M.A., Loepke A.W. (2016). Dosing and efficacy of intranasal dexmedetomidine sedation for pediatric transthoracic echocardiography: A retrospective study. Can. J. Anaesth. J. Can. D’anesthésie.

[90] Tenney J.R., Miller J.W., Rose D.F. (2019). Intranasal Dexmedetomidine for Sedation During Magnetoencephalography. J. Clin. Neurophysiol..

[91] Behrle N., Birisci E., Anderson J., Schroeder S., Dalabih A. (2017). Intranasal Dexmedetomidine as a Sedative for Pediatric Procedural Sedation. J. Pediatr. Pharmacol. Ther..

[92] Li L., Zhou J., Yu D., Hao X., Xie Y., Zhu T. (2020). Intranasal dexmedetomidine versus oral chloral hydrate for diagnostic procedures sedation in infants and toddlers: A systematic review and meta-analysis. Medicine.

[93] Zhang W., Wang Z., Song X., Fan Y., Tian H., Li B. (2016). Comparison of rescue techniques for failed chloral hydrate sedation for magnetic resonance imaging scans—Additional chloral hydrate vs. intranasal dexmedetomidine. Paediatr. Anaesth..

[94] Ghai B., Jain K., Saxena A.K., Bhatia N., Sodhi K.S. (2017). Comparison of oral midazolam with intranasal dexmedetomidine premedication for children undergoing CT imaging: A randomized, double-blind, and controlled study. Paediatr. Anaesth..

[95] Mukherjee A., Das A., Basunia S.R., Chattopadhyay S., Kundu R., Bhattacharyya R. (2015). Emergence agitation prevention in paediatric ambulatory surgery: A comparison between intranasal Dexmedetomidine and Clonidine. J. Res. Pharm. Pract..

[96] Cozzi G., Monasta L., Maximova N., Poropat F., Magnolato A., Sbisa E., Norbedo S., Sternissa G., Zanon D., Barbi E. (2017). Combination of intranasal dexmedetomidine and oral midazolam as sedation for pediatric MRI. Paediatr. Anaesth..

[97] Bua J., Massaro M., Cossovel F., Monasta L., Brovedani P., Cozzi G., Barbi E., Demarini S., Travan L. (2018). Intranasal dexmedetomidine, as midazolam-sparing drug, for MRI in preterm neonates. Paediatr. Anaesth..

[98] Patel V., Singh N., Saksena A.K., Singh S., Sonkar S.K., Jolly S.M. (2018). A comparative assessment of intranasal and oral dexmedetomidine for procedural sedation in pediatric dental patients. J. Indian Soc. Pedod. Prev. Dent..

[99] Boriosi J.P., Eickhoff J.C., Hollman G.A. (2019). Safety and Efficacy of Buccal Dexmedetomidine for MRI Sedation in School-Aged Children. Hosp. Pediatrics.

[100] Xu D., Xiu M., Zhang X., Zhu P., Tian L., Feng J., Wu Y., Zhao Z., Luan H. (2018). Effect of dexmedetomidine added to ropivicaine for caudal anesthesia in patients undergoing hemorrhoidectomy: A prospective randomized controlled trial. Medicine.

[101] Tu Z., Tan X., Li S., Cui J. (2019). The Efficacy and Safety of Dexmedetomidine Combined with Bupivacaine on Caudal Epidural Block in Children: A Meta-Analysis. Med. Sci. Monit..

[102] Lundblad M., Marhofer D., Eksborg S., Lonnqvist P.A. (2015). Dexmedetomidine as adjunct to ilioinguinal/iliohypogastric nerve blocks for pediatric inguinal hernia repair: An exploratory randomized controlled trial. Paediatr. Anaesth..

[103] Abdulatif M., Fawzy M., Nassar H., Hasanin A., Ollaek M., Mohamed H. (2016). The effects of perineural dexmedetomidine on the pharmacodynamic profile of femoral nerve block: A dose-finding randomised, controlled, double-blind study. Anaesthesia.

[104] Andersen J.H., Grevstad U., Siegel H., Dahl J.B., Mathiesen O., Jaeger P. (2017). Does Dexmedetomidine Have a Perineural Mechanism of Action When Used as an Adjuvant to Ropivacaine?: A Paired, Blinded, Randomized Trial in Healthy Volunteers. Anesthesiology.

[105] Vorobeichik L., Brull R., Abdallah F.W. (2017). Evidence basis for using perineural dexmedetomidine to enhance the quality of brachial plexus nerve blocks: A systematic review and meta-analysis of randomized controlled trials. Br. J. Anaesth..

[106] Jung H.S., Seo K.H., Kang J.H., Jeong J.Y., Kim Y.S., Han N.R. (2018). Optimal dose of perineural dexmedetomidine for interscalene brachial plexus block to control postoperative pain in patients undergoing arthroscopic shoulder surgery: A prospective, double-blind, randomized controlled study. Medicine.

[107] Bharti N., Sardana D.K., Bala I. (2015). The Analgesic Efficacy of Dexmedetomidine as an Adjunct to Local Anesthetics in Supraclavicular Brachial Plexus Block: A Randomized Controlled Trial. Anesth. Analg..

[108] Dutta A., Sethi N., Sood J., Panday B.C., Gupta M., Choudhary P., Puri G.D. (2019). The Effect of Dexmedetomidine on Propofol Requirements During Anesthesia Administered by Bispectral Index-Guided Closed-Loop Anesthesia Delivery System: A Randomized Controlled Study. Anesth. Analg..

[109] Wu X., Hang L.H., Wang H., Shao D.H., Xu Y.G., Cui W., Chen Z. (2016). Intranasally Administered Adjunctive Dexmedetomidine Reduces Perioperative Anesthetic Requirements in General Anesthesia. Yonsei Med. J..

[110] Nagoshi M., Reddy S., Bell M., Cresencia A., Margolis R., Wetzel R., Ross P. (2018). Low-dose dexmedetomidine as an adjuvant to propofol infusion for children in MRI: A double-cohort study. Paediatr. Anaesth..

[111] Di M., Yang Z., Qi D., Lai H., Wu J., Liu H., Ye X., ShangGuan W., Lian Q., Li J. (2018). Intravenous dexmedetomidine pre-medication reduces the required minimum alveolar concentration of sevoflurane for smooth tracheal extubation in anesthetized children: A randomized clinical trial. BMC Anesthesiol..

[112] Zhang X., Wu J., Wang L., Li W. (2018). Dexmedetomidine facilitates extubation in children who require intubation and respiratory support after airway foreign body retrieval: A case-cohort analysis of 57 cases. J. Anesth..

[113] Wang F., Zhong H., Xie X., Sha W., Li C., Li Z., Huang Z., Chen C. (2018). Effect of intratracheal dexmedetomidine administration on recovery from general anaesthesia after gynaecological laparoscopic surgery: A randomised double-blinded study. BMJ Open.

[114] Tsiotou A.G., Malisiova A., Kouptsova E., Mavri M., Anagnostopoulou M., Kalliardou E. (2018). Dexmedetomidine for the reduction of emergence delirium in children undergoing tonsillectomy with propofol anesthesia: A double-blind, randomized study. Paediatr. Anaesth..

[115] Kim H.S., Byon H.J., Kim J.E., Park Y.H., Lee J.H., Kim J.T. (2015). Appropriate dose of dexmedetomidine for the prevention of emergence agitation after desflurane anesthesia for tonsillectomy or adenoidectomy in children: Up and down sequential allocation. BMC Anesthesiol..

[116] Hauber J.A., Davis P.J., Bendel L.P., Martyn S.V., McCarthy D.L., Evans M.C., Cladis F.P., Cunningham S., Lang R.S., Campbell N.F. (2015). Dexmedetomidine as a Rapid Bolus for Treatment and Prophylactic Prevention of Emergence Agitation in Anesthetized Children. Anesth. Analg..

[117] Keles S., Kocaturk O. (2017). The Effect of Oral Dexmedetomidine Premedication on Preoperative Cooperation and Emergence Delirium in Children Undergoing Dental Procedures. BioMed Res. Int..

[118] Trivedi S., Kumar R., Tripathi A.K., Mehta R.K. (2016). A Comparative Study of Dexmedetomidine and Midazolam in Reducing Delirium Caused by Ketamine. J. Clin. Diagn. Res..

[119] Song Y., Shim J.K., Song J.W., Kim E.K., Kwak Y.L. (2016). Dexmedetomidine added to an opioid-based analgesic regimen for the prevention of postoperative nausea and vomiting in highly susceptible patients: A randomised controlled trial. Eur. J. Anaesthesiol..

[120] Chrysostomou C., Schulman S.R., Herrera Castellanos M., Cofer B.E., Mitra S., da Rocha M.G., Wisemandle W.A., Gramlich L. (2014). A phase II/III, multicenter, safety, efficacy, and pharmacokinetic study of dexmedetomidine in preterm and term neonates. J. Pediatrics.

[121] Franciscovich C.D., Monk H.M., Brodecki D., Rogers R., Rintoul N.E., Hedrick H.L., Ely E. (2020). Sedation Practices of Neonates Receiving Extracorporeal Membrane Oxygenation. ASAIO J..

[122] Sperotto F., Mondardini M.C., Vitale F., Daverio M., Campagnano E., Ferrero F., Rossetti E., Vasile B., Dusio M.P., Ferrario S. (2019). Prolonged sedation in critically ill children: Is dexmedetomidine a safe option for younger age? An off-label experience. Minerva Anestesiol..

[123] Dersch-Mills D.A., Banasch H.L., Yusuf K., Howlett A. (2019). Dexmedetomidine Use in a Tertiary Care NICU: A Descriptive Study. Ann. Pharmacother..

[124] Puthoff T.D., Shah H., Slaughter J.L., Bapat R. (2018). Reduction of Analgesia Duration after Tracheostomy during Neonatal Intensive Care: A Quality Initiative. Pediatr. Qual. Saf..

[125] Weatherall M., Aantaa R., Conti G., Garratt C., Pohjanjousi P., Lewis M.A., Moore N., Perez-Gutthann S. (2017). A multinational, drug utilization study to investigate the use of dexmedetomidine (Dexdor(R)) in clinical practice in the EU. Br. J. Clin. Pharmacol..

[126] Grant M.J., Schneider J.B., Asaro L.A., Dodson B.L., Hall B.A., Simone S.L., Cowl A.S., Munkwitz M.M., Wypij D., Curley M.A. (2016). Dexmedetomidine Use in Critically Ill Children With Acute Respiratory Failure. Pediatr. Crit. Care Med..

[127] Piastra M., Pizza A., Gaddi S., Luca E., Genovese O., Picconi E., De Luca D., Conti G. (2018). Dexmedetomidine is effective and safe during NIV in infants and young children with acute respiratory failure. BMC Pediatr..

[128] Ghimire L.V., Chou F.S. (2018). Efficacy of prophylactic dexmedetomidine in preventing postoperative junctional ectopic tachycardia in pediatric cardiac surgery patients: A systematic review and meta-analysis. Paediatr. Anaesth..

[129] Flores-Gonzalez J.C., Estalella-Mendoza A., Lechuga-Sancho A.M., Hernandez-Gonzalez A., Rubio-Quinones F., Rodriguez-Campoy P., Saldana-Valderas M. (2017). Supraventricular tachycardia after withdrawal of prolonged dexmedetomidine infusion in a paediatric patient without heart disease. J. Clin. Pharm. Ther..

[130] Burns J., Jackson K., Sheehy K.A., Finkel J.C., Quezado Z.M. (2017). The Use of Dexmedetomidine in Pediatric Palliative Care: A Preliminary Study. J. Palliat. Med..

[131] De Zen L., Marchetti F., Barbi E., Benini F. (2018). Off-label drugs use in pediatric palliative care. Ital. J. Pediatr..

[132] De Zen L.D.R., Robazza M., Barbieri F., Campagna M., Vaccher S., Barbi E., Dall′Amico R. (2020). Home intranasal dexmedetomidine in a child with an intractable sleep disorder. J. Pediatr. Pharmacol. Ther..

[133] De Zen L., Della Paolera S., Del Rizzo I., Taucar V., Skabar A., Barbi E. (2020). Home Intranasal Dexmedetomidine for Refractory Dystonia in Pediatric Palliative Care. J. Pain Symptom Manag..

[134] Li B.L., Yuen V.M., Zhang N., Zhang H.H., Huang J.X., Yang S.Y., Miller J.W., Song X.R. (2019). A Comparison of Intranasal Dexmedetomidine and Dexmedetomidine Plus Buccal Midazolam for Non-painful Procedural Sedation in Children with Autism. J. Autism Dev. Disord..

[135] Carlone G., Trombetta A., Amoroso S., Poropat F., Barbi E., Cozzi G. (2019). Intramuscular Dexmedetomidine, a Feasible Option for Children With Autism Spectrum Disorders Needing Urgent Procedural Sedation. Pediatr. Emerg. Care.

[136] Lubisch N., Roskos R., Berkenbosch J.W. (2009). Dexmedetomidine for procedural sedation in children with autism and other behavior disorders. Pediatr. Neurol..

[137] Abulebda K., Louer R., Lutfi R., Ahmed S.S. (2018). A Comparison of Safety and Efficacy of Dexmedetomidine and Propofol in Children with Autism and Autism Spectrum Disorders Undergoing Magnetic Resonance Imaging. J. Autism Dev. Disord..

[138] Stuker E.W., Eskander J.P., Gennuso S.A. (2018). Third time’s a charm: Oral midazolam vs intranasal dexmedetomidine for preoperative anxiolysis in an autistic pediatric patient. Paediatr. Anaesth..

[139] Mason K.P., Lubisch N., Robinson F., Roskos R., Epstein M.A. (2012). Intramuscular dexmedetomidine: An effective route of sedation preserves background activity for pediatric electroencephalograms. J. Pediatr..

